# The Unity or Duality of the Syphilitic Virus

**Published:** 1871-10

**Authors:** Edmund Andrews

**Affiliations:** Professor of Principles and Practice of Surgery in Chicago Medical College; No. 6, 16th Street, Chicago


### CHICAGO MEDICAL EXAMINER:

N. S. DAVIS, M. D., EDITOR.

F. H DAVIS, M. D., ASSISTANT EDITOR.

VOL. XII. OCTOBER, 1871. NO. 10.

### ORIGINAL CONTRIBUTIONS.

### THE UNITY OR DUALITY OF THE SYPHILITIC VIRUS.

BY EDMUND ANDREWS, M. D., Professor of Principles and Practice of Surgery in Chicago Medical College.

THE Franco-German war interrupted a scientific controversy on this subject, which had lasted with remarkable fluctuations for nearly forty years. The ancients do not seem to have had any such question before them until the great venereal epidemic which commenced at the seige of Naples, near the end of the 15th century, At that time the physicians seem to have recognized two diseases, but their successors gradually adopted the idea that all the venereal diseases were essentially one, which state of opinion continued without much discussion until 1831. At that time one of the most remarkable men ever enrolled in the ranks of surgery commenced a series of experiments upon venereal diseases by inoculations.

This man was Philip Ricord, Surgeon of the *Hôpital du Midi* in Paris. Ricord had an immense talent as an experimenter, and possessed a clear and powerful mode of stating his propositions. To these good traits he added a habit of bearing down opposition by a confident air, a domineering manner, and by offensive personalities applied to his opponents. His love of confident statements verged toward disregard of truth, leading him to assert as positive very much that was doubtful, and even to contradict his own previous statements, when the drift of his new arguments seemed to require it. By the help of these varied qualities he got himself looked upon as a hero, and may be said in a certain sense to have bound French scientific thought and dragged it by the heels behind his chariot. Having carried on his experimental inoculations with great ability for seven years, he brought out the results in 1838 in his famous work, *Traité Pratique des Maladies Vénériennes*, and repeated them in 1840, in annotations upon his translation of Hunter's *Treatise on Venereal Diseases.*

These works were of great value to the progress of syphilology, and created an immense enthusiasm. In the first treatise he set forth among many others, the following important doctrines.

First. A syphilitic virus does exist.

Second. There is but one syphilitic virus.

Third. The only constant and decisive symptom of chancre is not color, hardness, etc., but solely the secretion of pus with infecting qualities.

Fourth. The only vehicle of the poison is the pus, and secondary and tertiary symptoms are not contagious.

Ricord,therefore, commenced as an advocate of unicity,and asserted the doctrine with all that audacious positiveness which gave him such a control over the minds of his disciples. One of his doctrines, however, contained a seed which ultimately germinated and overturned his own position. It was contained in these words: "An indurated chancre, or constitutional syphilis is only communicable once in a person's life-time."

In the mean time, Ricord's doctrine that secondary syphilis was not contagious, was being undermined. Wallace, a very thorough and careful investigator in Dublin, had observed many instances of secondary contagion, and had demonstrated by experiments that secondary disease was certainly transmissible. (London Lancet, Vols. 31 and 32.) Ricord and his disciples, however, entirely ignored all investigations which had not been made in Paris, and went on asserting the non-contagiousness of secondary syphilis.

The matter was taken up, however, by Waller, of Prague, who published in the *Prague Quarterly* in 1851, a series of observations and experiments, some of which were repeated by Wallace. In these cases it was shown over and over again in the most careful manner that healthy children took syphilis from nurses, having only secondary disease, that nurses took it from children in the same stage, and wives from their husbands. Experiments showed that pus from condylomata lata, and even the blood of secondary syphilitics, would by inoculation reproduce the disease on healthy persons. The German surgeons, Bärensprung, of Berlin, Rinecker and Lindmann, all followed suit with separate experiments, abundantly establishing the same doctrine. Ricord at first attempted to oppose these results by his customary fusilade of sarcasm and personal abuse, but he was shortly silenced by a connonade of facts and compelled to acknowledge his blunder. The French surgeons, who had made a sort of demi-god of Ricord, were deeply chagrined at his defeat and scarcely knew what to do with themselves. Residents in Paris describe them as having been about as inconsolable, as they were afterwards when the Germans took the city.

But the prestige which the French lost in one way they regained in another. Bassereau, a disciple of Ricord, in 1852 brought out his *Traité des affections de la peau symptomatiques de la syphilis*, in which the great doctrine of dualism was, in our day, first distinctly propounded. He made a large number of careful observations and experiments, and showed by what he called "*confrontations*,"

that is, the comparison of each case with the one from which its contagion was derived, that there are two distinct diseases, one chancroid, which is a purely local disease, has a suppurating bubo and never infects the constitution; the other the hard, or infecting chancre, which always infects the constitution. He called the first "*chancre with suppurating bubo*," and the second "*chancre which precedes constitutional infection.*" In 1854, Clerc, of Paris, came out in advocacy of the same doctrine, calling the soft chancre "*chancroid*," and maintaining that there were two distinct diseases, and that each propagated its own kind exclusively. Rollet, of Lyons, and others, next bronght out the theory of "*mixed chancre*," showing that when both kinds of virus were inoculated in one spot, a mixed form of ulcer ensued, having the characteristics of both predecessors.

The famous doctrine of dualism was thus fully constructed. Ricord himself adopted it, though it required him to abandon another of his original propositions, (the one which asserted that there was but one syphilitic virus,) and it soon swept over the whole of Europe. In 1861, Dr. Bumstead, of New York, published a work on venereal diseases, in which he advocated the new European ideas, and became a prominent means of causing dualism to be generally accepted in this country.

The dualistic theory thus gained a great and almost universal triumph, though a few resolute unicists still stood out. Prof. Gross, of Philadelphia, never surrendered; Vidal, in France, continued to fight the new theory violently, while in Germany, Hebra and Michælis, and in Scandinovia, Bœck, Bidenkap, and Danielson, stubbornly held their ground.

Bœck, of Christiania, was engaged extensively in the cure of secondary disease by syphilization. In this treatment he inoculated his patients over and over again with virus from syphilitic sores. The patient being already under the influence of disease, of course the product of inoculation upon him was only soft chancre, but Bœck, who was a unicist, believed that all syphilitic sores were of the same nature, and that by making them in large numbers on the patient, he hastened on the progress of the disease, and got the patient much sooner through it into perfect health. In the course of his experiments he discovered that the statements of the dualists, first put forth by Bassereau, that each kind of chancre propogated only its own species, was false. He proved beyond dispute that though the secretion of a hard chancre in a non-inflamed state produced no effect on a syphilitic patient, yet the same sore, when irritated until it secreted a thick pus, gave origin to matter which would produce soft chancres. In fact, Bœck used habitually to obtain new supplies of soft chancre virus for his patients by irritating either hard chancres or secondary sores, such as mucous patches, until they suppurated freely.

Bidenkap, the pupil of Bœck, and Köbner of Germany instituted careful experiments by inoculation, and arrived with more precision at similar conclusions, which may be stated as follows:

1. The secretion of an unirritated pure hard chancre produces pure hard chancre on persons who have never had constitutional syphilis.

2. On those who have already had the disease it produces nothing.

3. If now you irritate the same hard chancre until it secretes a thick pus, it will produce on a syphilitic man genuine soft chancres, which can be repeated upon him a great number of times. In short, the virus of soft chancre comes from an inflamed hard one.

These experiments shook the foundations of dualism like an earthquake, and many eminent men abandoned the theory, declaring it no longer tenable, while its remaining adherents were taken by surprise and unable to see exactly how they were to defend themselves. Of course the unicists were jubilant over their remarkable victory.

Such was the position of the contending parties when the thunder clouds of the Franco-German war rolled across the field, and compelled a cessation of hostilities. On reviewing the whole matter, it is evident that some of the most confident assertions of the dualists are overthrown, and it must be confessed that the whole theory is in a dilapidated condition, yet in some points it may still be defensible. We must apparently admit that the virus of soft chancre may be produced at will by irritating a hard chancre, but are we on that account sure of the identity of the two? Being aware that one sore may give exit to several chemical compounds, are we sure that it may not give origin to two poisons? The clinical history of cases, would suggest the conclusion that if a person who is insusceptible to hard chancre and to constitutional syphilis in consequence of having gone through them—if such a person were inoculated with a mixed virus from an inflamed hard chancre, his system, proof against the virus of the hard sore, would propagate and transmit to others only the soft one, thus producing pure and unmixed soft chancres, free from all contamination of the hard ones. If facts should prove such a result, they would seem to establish a kind of modified dualism.

More experiments are needed to settle this point, but the following certainly look in that direction.

1. Bidenkap reports a girl in hospital and free from syphilis. Being in a ward where patients were being syphilized, she by means of a pin inoculated herself in sport from one of the soft chancres of a patient undergoing that treatment. She had a regular soft chancre, but though carefully watched, and examined once a week for two years, she had no indurated sore, nor constitutional disease. At the end of that time she got syphilis from a new source, which was shortly followed by constitutional disease. Here certainly seemed to be a case of soft chancre which was quite free from the virus of the hard chancre.

2. Another girl did the same thing, and the ulcers were repeated upon her some twenty times by Bidenkap. Although kept under observation nearly a year she showed no secondary symptoms.

3. Danielson of Christiania tried syphilization on leprosy. The leprosy was not cured, but the following important case occurred. One of the lepers was inoculated for six months from soft chancres, having nearly three hundred soft chancres in all. Up to this time, no true syphilis showed itself. He was then inoculated from a hard chancre, and in the usual time had constitutional syphilis.

4. Auzias—Turenne proved that the inferior animals could have soft chancre, but not constitutional syphilis. The surgeon Diday, who had never had syphilis, inoculated a chancre on the head of a cat. From this he inoculated his own penis, which produced a soft chancre and an obstinate suppurating bubo. No constitutional syphilis followed.

5. Robert de Weltz in the same way inoculated a monkey's ear, and from the sore resulting inoculated his own arm. He had a troublesome ulcer, and suppurating glands, but no constitutional syphilis.

It can not be said that these experiments fully settle the question, but I think the following conclusions will be ultimately arrived at.

1. There are two kinds of virus.

2. A pure, uninflamed, hard chancre gives out only the hard chancrous virus, but if it be irritated until it secretes thick pus freely, it will yield both kinds.

3. If this double virus be inserted into a healthy animal, or a man insusceptible to hard chancre and to constitutional syphilis, the soft chancre only is produced.

4. If the virus of this last soft chancre be inoculated into any person whatever, it will probably produce only pure soft chancre, and no constitutional disease.

If these conclusions shall be ultimately sustained, dualism will after all win a sort of victory, the two kinds of virus will be proved, but it will be a barren conquest, because the two poisons are so intimately connected, that the surgeon can hardly ever rely on the absence of the poison of the hard chancre, and hence he will be compelled to practice very much like an unicist.

The fluctuations of this remarkable controversy are adapted to arouse indignation in a thinking mind. What is the use of science in the hands of men who contrive, not how to bring out solid and permanent truth, but only some startling conclusions upon which to base a reputation? Who are these pseudo-great men, to whom we have given the highest honors of science for forty years, for telling us, first that the syphilitic virus is single, then that it is undoubtedly double, then again that it is single, and finally that they don't know which it is? Would any blockhead have done worse by simple guess work? There is only one remedy for this foolery, and that is to leave off honoring men for half made discoveries, and to scourge with the lash of criticism and contempt all efforts to place crude and ill sustained opinions in the rank of ascertained truths.

No. 6, 16th Street, Chicago.

